# Non-coding yet non-trivial: a review on the computational genomics of lincRNAs

**DOI:** 10.1186/s13040-015-0075-z

**Published:** 2015-12-22

**Authors:** Travers Ching, Jayson Masaki, Jason Weirather, Lana X. Garmire

**Affiliations:** 1 Molecular Biosciences and Bioengineering Graduate Program, University of Hawaii at Manoa, Honolulu, HI 96822 USA; 2 Epidemiology Program, University of Hawaii Cancer Center, Honolulu, HI 96813 USA; 3 Laboratory of Immunology and Signal Transduction, Chaminade University of Honolulu, Honolulu, HI 96816 USA; 4 Department of Internal Medicine, University of Iowa, Iowa City, IA 52242 USA

## Abstract

Long intergenic non-coding RNAs (lincRNAs) represent one of the most mysterious RNA species encoded by the human genome. Thanks to next generation sequencing (NGS) technology and its applications, we have recently witnessed a surge in non-coding RNA research, including lincRNA research. Here, we summarize the recent advancement in genomics studies of lincRNAs. We review the emerging characteristics of lincRNAs, the experimental and computational approaches to identify lincRNAs, their known mechanisms of regulation, the computational methods and resources for lincRNA functional predictions, and discuss the challenges to understanding lincRNA comprehensively.

## Introduction

The mainstream focus of biomedical research has been in elucidating the functions and interactions among proteins within the cell. In line with the central dogma of molecular biology, RNAs were once perceived as the intermediary for protein production and the archaic precursor molecule of DNA. However, RNAs are transcribed from more than 85 % of genomic regions [1], whereas proteins are only encoded in less than 3 % of human genome sequences [2]. This leaves a mysterious knowledge gap in either the efficiency of cellular transcription to translation or a foundational misunderstanding in gene expression regulation and RNA function. It was thought that RNAs had limited but essential and evolutionarily common roles of basic cell machinery such as tRNA, rRNA, and mRNA. The few examples of functional RNAs or RNAs with enzymatic-like activity, were considered as evolutionary remnants [3]. For a long period of time, non-coding RNA (ncRNA) transcripts were believed to be by-products derived from mRNA degradation or nonspecific polymerase activity, and therefore termed “transcriptional noise” [4].

It is now becoming evident that ncRNAs are responsible for many aspects of gene regulation. Some small non-coding RNAs, such as microRNAs, siRNAs, snRNAs, snoRNAs, exRNAs and piRNAs, have been well categorized over the past decade. However, long noncoding RNAs (lncRNAs) remained relatively unexplored due to the challenges of computational prediction under poor sequence conservation and low homology within the set of lncRNAs. Some of these challenges have been addressed by the revolutionary inventions of next generation sequencing (NGS) and its applications, such as RNA-Seq, which captures whole transcriptome data, including lncRNAs. Among the human lncRNAs, tens of thousands of long intergenetic noncoding RNAs (lincRNAs) have been discovered in the genomic regions outside of the well-studied coding genomic regions, and they show many intriguing properties, such as associations with various human diseases, tissue-specific expression, and expression changes during development. Consequently, attributing organism complexity to the hidden regulation of lincRNAs is a fascinating new area of research. Here, we review the emerging characteristics of lincRNAs; the experimental and computational approaches to identifying lincRNAs and their mechanisms of regulation; the challenges in computational predictions; and the resources still required to advance our understanding of lincRNA-related genomic regulation.

## Review

### Emerging characteristics of lincRNAs

LincRNAs are a putatively heterogeneous group, conventionally defined as ncRNA transcripts of more than 200 bp located in regions with no overlap to any known protein-coding genes. According to Lncipedia, a comprehensive lncRNA database, high-throughput studies of transcriptome data have catalogued over 111,000 lncRNA transcripts, with roughly 50 % coming from intergenic regions [5]. The majority of lincRNAs are thought to be transcribed from RNA polymerase II, and are therefore usually modified by post-transcriptional 5′ capping and 3′ polyadenylation [6]. Surprisingly, lincRNAs show ribosome occupancy similar to the 5′UTRs of protein coding genes [7]. What differentiates lincRNAs from protein coding genes seems to be the lack of release upon encountering a stop codon in the lincRNA sequences [7]. Therefore, polyadenlyation and 5′ capping are not necessarily markers of protein coding functionality. However, lincRNAs show a markedly higher degree of tissue-specific [8] and disease specific expression [9], suggesting some biological function.

LincRNA expression is generally much lower than protein coding genes, with a few exceptions such as the XIST lincRNA [10]. For some lincRNAs, even just a few or a single transcript exist in a cell, determined by RNA-Seq data [10]. However, rather than being spurious by-products of non-specific RNA transcription, the expression levels of lincRNAs in any given cell are precisely coordinated throughout the tissue, and dynamic through the course of development [11]. Researchers have detected differential expression of lincRNA in a range of tissues, diseases, and specific cellular responses. Efforts have been made to take advantage of these properties of lincRNAs for translational and clinical applications, such as disease biomarkers [12].

Another unique feature of lincRNAs is the low sequence conservation. LincRNAs exhibit 22–25 % of conserved bases under purifying selection, compared to 77 % in protein coding sequences. However, they are considerably more conserved than introns, which have 7 % conservation [13]. Under the assumption that sequence conservation reflects biological significance, the high genomic sequence variability in lincRNAs was the initial basis to call them “junk RNAs”. Unlike proteins, where evolutionary conservation correlates highly with functional importance, lincRNAs seem to be under different selective pressures. Many lincRNA are predicted to have secondary structure and may therefore act in a sequence independent manner [14]. Consequently, there may be a greater functional importance on molecular 3D conformation over the primary sequence. This is supported by a recent global study of genetic variants in human lincRNAs in association with diseases, where single nucleotide polymorphisms (SNPs) in evolutionarily conserved regions of lincRNAs had significant effects on predicted secondary structure [15].

### Genome-wide detection of lincRNAs

Chromatin immunoprecipitation sequencing (ChIP-Seq) is an NGS method that has allowed the discovery of global genomic binding sites of DNA-interacting proteins, such as transcription factors and histones. Using ChIP-Seq signatures of histone 3 lysine 4 tri-methylation (H3K4me3) and histone 3 lysine 36 tri-methylation (HK36me3), or so called “K4-K36” clusters, Guttman et al. detected approximately 1700 transcriptional units >5 kb among four mouse cell lines, which were confirmed by tiling microarrays, PCR and northern blots [16]. This type of chromatin signature was later applied to human cell lines to identify lincRNAs and was shown that along with HOTAIR, 20 % of lincRNAs were associated with the Polycomb repressive complexes 2 (PRC2) [4]. ChIP-Seq has also been applied to the detection of RNA pol II occupancy to identify lincRNAs in mouse macrophages upon endotoxin stimulation [17]. The authors found that 70 % of extragenic polymerase II peaks were associated with genomic regions with a canonical chromatin signature of enhancers.

Clearly, decisions made during the library preparation phase of an RNA-seq experiment will affect lincRNA measurements. Since many but not all lincRNA transcripts are poly-adenylated [18], the decision to select poly-adenylated RNAs or to use ribodepletion methods should be made with care. Yang et al. [19] state that approximately 20 % of transcripts are non-poly adenylated, suggesting that ribo-depletion methods are necessary to gain a more comprehensive picture of the transcriptome. In addition, Yang et al. find that some transcripts, such as the Malat1 lincRNA are bimorphic, meaning they exist in poly-A(+) and poly-A(−) configurations. Thus, ribo-depletion and poly-A selection methods could provide complementary information on the relative proportions of poly-adenylation of transcripts. Moreover, the adoption of strand-specific sequencing protocols provides a means of making more detailed annotations of lncRNAs, especially the antisense lncRNAs [20]. Nevertheless, even without strand information, RNA-seq has proven useful for the identification of lincRNAs. For example, Cabili et al. analysed lincRNAs in 24 tissues and mapped out nearly 9000 lincRNAs coupled to expression profile information [8].

Not all NGS methods are ideal for identifying the precise boundaries of lincRNAs. ChIP-Seq using antibodies against RNA polymerases can only provide a rough estimation of transcription location but not the precise boundaries of transcripts [17]. RNA-Seq may also have trouble to detect isoforms and their exact start and end sites, as the cDNA is randomly fragmented, and accumulated from all isoforms within a given genomic loci [21]. Moreover, if RNA-Seq is conducted by a poly-A enriched approach, the internal bias against 5′ ends make it difficult to map out the exact start sites of a transcript. However, some other NGS methods have been adopted to overcome this problem. For example, cap analysis gene expression (CAGE) tag sequencing has been used to aid the identification of transcription start sites in human cells [18], and 3′-end sequencing (3SEQ) has also been used in a zebrafish model to aid the determination of the 3′ bounds of lincRNA transcripts [22]. Additionally, tiling arrays that enable direct observation of lincRNAs transcript exons have been used to detect gene boundaries and alternative splicing. For example, Tahira et al. sampled intergenic and intronic ESTs from over one million ESTs from The Cancer Genome Project to develop a custom microarray, and subsequently identified lincRNAs differentially expressed between primary and metastatic pancreatic cancers [23].

### Computational methods to predict lincRNAs

Most computational studies of lincRNAs rely on RNA-Seq results initially, with quality-control filtering steps to remove reads arising from spurious background noise [24]. Additional steps should be taken involving the removal of protein coding genes and small non-coding RNAs such as microRNAs. Methods to do such removals include ORF detection [1, 9, 16, 25], BLAST to identify homologs of protein coding genes [25], domain based searches such as Pfam [9, 25], and predictions of coding potential based on nucleotide substitution frequencies given sequences from multiple species. The Coding Potential Calculator (CPC) [26] and iSeeRNA [27] programs are popular choices in determining coding potential. However, the extent to which some lincRNAs may be hosts of smaller RNA species such as microRNAs requires further study [28]. Another selection criterion is the number of exons in a transcript. Most of the exons (about 80 % in human) are less than 200 bp [29], the minimum length requirement of lincRNA by definition. Transcripts with only one exon are less likely to be lincRNAs. Additionally, the number of exons can be used as an indicator of transcript quality. Multi-exonic transcripts are less likely to result from spurious transcription and genomic noise. The presence of introns is also indicative of robust and consistent transcription boundaries. Introns have less frequent terminal repeats and transposable elements in comparison to intergenic regions, suggesting that lincRNAs have additional conservation in splicing [30]. Finally, the axiomatic length-based filter, 200 bp, eliminates any non-coding sequences that fall into the current small RNA categories [31]. The filtering steps described above are often implemented through a pipeline with a series of cut-offs or a decision tree to interrogate multiple features involved in classifying lincRNAs [24].

In recent years, machine learning based classification approaches have been used to detect lincRNAs [17, 27, 32–34]. For example, iSeeRNA interrogated coding potential based on a variety of factors mentioned above, in addition to nucleotide composition. It was trained to differentiate protein coding genes and lncRNAs with an area under the curve (AUC) of 0.99 [27]. LncRNA-MFDL is another tool that uses a deep learning method and the fusion of multiple features to classify lincRNAs with an accuracy of 97.1 % [34].

### lincRNA databases

LincRNAs identified from exploratory studies are a valuable resource for accumulating information about these relatively unknown transcripts. Information such as location, splice junction, and tissue specificity are important features. There are quite a few specialized databases that provide comprehensive annotations for lincRNAs or lncRNAs. These include The Broad Institute’s Human Body Map project [4], NONCODE [35] and Lncipedia [5]. Other large gene annotation sets such as GENCODE [36, 37], UCSC’s known genes [38] or Rfam [39] RNA family databases are not specific to non-coding RNAs, but nevertheless contain large sets of annotations and information on lincRNAs.

The UCSC ENCODE project provides a feature-rich resource to describe the transcriptional landscape in a variety of tissues from the GENCODE database [40]. The Ensembl Geneome Browser is another resource that identifies and annotates transcripts within their large database using transcriptional evidence as well as chromatin mark-ups [36]. The Ensembl project uses the GENCODE database, and contributes multiple sources to GENCODE through an automated annotation pipeline in combination with the large Havana annotation by the Sanger Institute [36]. While GENCODE is one of the most comprehensive databases for mammalian species, it does not include lincRNAs found by RNA-Seq *ab initio* alignment methods, such as those in the Human Body Map. Neither is it as comprehensive as specialized databases.

More specialized lncRNA databases, such as NONCODE and Lncipedia, enumerate a much larger number of lncRNAs (Table 1). These databases have been created to facilitate functional analyses by integrating multiple data sources such as expression, chromatin markups, microRNA binding sites and mutational data with known lncRNAs. Not surprisingly, the overlap of those data sets can differ greatly, largely due to the selection criteria of particular lncRNAs or the tissue origins where lincRNAs were initially detected.

**Table 1:** Summary of lncRNA/lincRNA databases

Project Name	Species	Purpose
Human Body Map	Human	A reference set of lincRNAs
ChIPBase	Various (incl. Human and Mouse)	A resource for lncRNA transcriptional regulation and expression profiles of ncRNA (lncRNA, microRNAs, etc.)
NONCODE	Various (incl. Human and Mouse)	A large lncRNA database integrating various databases and references
lncRNAdb	Various (incl. Human and Mouse)	A database of lncRNAs having biological function or regulatory roles
ncRNA expression database (NRED)	Human and Mouse	Expression database for human and mouse lncRNAs
LNCipedia	Various (incl. Human and Mouse)	A large database of lncRNA transcripts and annotation
LncRNADisease	Human	A database of lncRNAs associated with human diseases
DIANA-LncBase	Human and Mouse	A database of experimentally verified and predicted microRNA targets on lncRNAs
lncRNA2Target	Human and Mouse	A collection of lncRNA knockout experiments and downstream regulation
starBase 2.0	Human, Mouse and C. elegans	A collection of lncRNA and predicted microRNA targets; lncRNA expression profiles from TCGA data
lncRNAMap	Human	A resource for exploring lncRNA expression profiles and interaction with small RNAs (siRNA, microRNAs, etc.)
lncRNAWiki	Human	An open wiki style lncRNA database
MONOCLdb	Mouse	A mouse noncoding database detailing functional enrichment of lncRNA in response to respiratory disease caused by influenza and SARS-CoV
lncRNome	Human	A searchable database for long noncoding RNAs in humans and various properties, such as predicted structure, SNPs and epigenetic modifications
PLncDB	Arabidopsis thaliana	A database dedicated to A. thaliana plant lncRNA transcriptome, including information on epigenetic modification
Functional lncRNA Database	Human, Mouse and Rat	A database of experimentally validated functional lncRNAs
lnCeDB	Human	A database of lncRNA acting as ceRNA
Linc2GO	Human	A database of lncRNA acting as ceRNA and biological processes based on GO annotation
lncRNASNP	Human and Mouse	A database cataloging micro-RNA interactions and SNPs in lncRNAs and their impact on secondary structure

### Genomic assays to study lincRNA regulations

Methods to elucidate the functions of individual lincRNAs have made much slower progress compared to large-scale genomic assays. In this section we survey the increasing number of genome-scale molecular interaction studies to investigate the cellular functions of lincRNAs.

Several genomic approaches have been reported to identify specific functions of lincRNAs. One popular technique is the protein-centric RNA immunoprecipitation (RIP), which selects a particular protein or a group of proteins to co-precipitate RNAs and determines functional relationships based on physical interactions [41]. This allows one to ascribe functions of the protein(s) with co-precipitated lincRNAs. For example, Shi et al. used RIP to identify novel functional lincRNAs involved in the regulation of TNF expression through binding to PRC2 [42], and found that PRC2 binds to thousands of RNA species. Thus, protein-centric methods focusing on PRC2 have provided us critical insights into the genome-wide regulation by lincRNAs [43].

Conversely, another approach is to purify certain RNA molecules and then capture the associated proteins (RNA-centric methods); the associated proteins can then be identified via mass-spectroscopy [41]. This approach works by complementary base pairing of the RNA sequence to oligonucleotide probes labelled with streptavidin or biotin [44]. However, in comparison to protein-centric methods where the RNA targets can be amplified by PCR, RNA-centric methods do not have a means of amplifying the protein targets. Therefore, RNA-centric methods work best when large quantities of protein are available [41].

Additionally, there have also been a handful of “DNA-centric” methods for studying lincRNAs. Methods that investigate DNA modification or the 3D structure of chromosomes have greatly advanced our understanding of gene regulation [45]. For example, Ma et al. developed a novel method called Dnase Hi-C that determines the interactions of lincRNA promoters with DNA enhancer regions [45]. Their method involves cross-linking nearby DNA strands, followed by DNAse I digestion, proximity ligation between the cross-linked strands and DNA sequencing. Rather than using restrictive enzyme (RE) as done in conventional Hi-C, which generates predictable and consistent fragment ends, DNase I produces a heterogeneous mixture of fragment ends that greatly improves the efficiency and resolution. They were able to fine-map cell specific 3D organization of 998 lincRNA promoters. They demonstrated that lincRNA expression is tightly controlled by complex mechanisms including super-enhancers and PRCs.

### Known functions and mechanisms of lincRNAs

Historically, lincRNAs have been shown to have a greater likelihood to be functionally associated with their nearest neighboring protein-coding genes. However, more recent analyses show that the expression correlation between a lincRNA and its closest coding gene is not statistically significant when compared to the correlation between two neighboring protein-coding genes [8, 46]. While complementary base pairing may be the mechanism of action for some small RNAs such as microRNAs, lincRNAs by their nature are unlikely to exert their regulatory function solely through sequence pairing. Instead, lincRNAs have been shown to mediate the interplay between many molecular species simultaneously [47]. LincRNAs affect gene expression by many different mechanisms - from chromatin remodelling and epigenetic regulation, to transcriptional, post-transcriptional, and protein-level control. So far, no unifying genome-wide theme has been found to explain all the complexities of lincRNA regulation. We review the handful of competing theories that attempt to address this problem.

#### LincRNAs involved in chromatin remodelling

Epigenetics is a vital means of DNA patterning to regulate gene expression [48]. PRCs exert gene silencing epigenetically by histone modifications and DNA chemical alterations such as methylation [43]. Recruitment of PRCs to certain genomic locations is mediated by specific lincRNAs. Thus, the differential expression of certain lincRNAs (such as HOTAIR) can lead to activation or deactivation of transcription on the genome [49]. The vital role of gene suppression due to lincRNAs has been implicated in the pathology of cancers, where dysregulation of individual lincRNAs release cell cycle control resulting in an increase in cell proliferation [50]. Complicating matters, thousands of lincRNAs were found bound by PRC2 within various cell types [4], suggesting the widespread interaction of lincRNAs with the epigenetic modification machinery.

#### LincRNAs as transcription co-factors

Many lincRNAs are known to act as transcription co-factors. In some cases, the act of transcription of a lincRNA may positively or negatively affect expression of nearby genes [51]. Dimitrova et al. showed that lincRNA-p21 acts as a transcriptional coactivator and was required for recruitment of ribonucleoproteins to promoter elements associated with pre-mRNA [52]. MALAT1 is also known to act as a transcription co-factor. This lincRNA is well characterized as one of the most highly expressed mammalian lincRNAs. It is also known to significantly affect the metastasic process in lung adenocarcinoma, by enhancing the expression of cell motility genes [53]. It was found that MALAT1 acts as a molecular scaffold to allow gene expression by promoting the interaction among unmethylated PRC2, E2F1 transcription factor, histone markers, and the other transcriptional co-activator complexes [54]. Interestingly, this protein sequestration mechanism of ncRNA is not unique to eukaryotes, and it also occurs in bacteria [55].

#### Competing endogenous RNA hypothesis of lincRNAs

The competing endogenous RNA (ceRNA) hypothesis is a theory that lncRNAs (including lincRNAs) regulate gene expression by acting as microRNA sponges [56]. The inhibition of specific mRNA translation is modulated by microRNA depletion through lncRNAs harboring microRNA binding sites. By effectively competing for the same microRNA, these lncRNAs exert a level of competitive inhibition. Based on this hypothesis, Liu et al. developed a database of lincRNAs that were predicted to have functional associations with protein-coding genes [57]. Some exemplary lincRNAs that function as ceRNAs are the HULC [58] and LINC-ROR [59]. HULC was shown to be the molecular sponge of a series of microRNAs including miR-372, which induces phosphorylation of CREB in liver cancer [60], and LINC-ROR shares the microRNA response elements with core transcription factors Oct4, Sox2, and Nanog and thus increases expression of these genes by competing for microRNAs [61]. Although some lincRNAs act as ceRNAs, it is unclear how prevalent this mechanism is among all lincRNAs.

#### LincRNAs as evolutionary reservoirs

While lincRNAs have less sequence conservation than protein-coding genes, they have a greater degree of secondary motif conservation compared to mRNAs [62]. These elements may explain the origins of lincRNAs, which provide a reservoir of evolutionarily constrained RNA motifs [62, 63] to supply extra genetic modules for evolutionary tinkering. It is also known that Retrotransposon and tandem repeat sequences are more common within lincRNAs compared to protein-coding genes [64]. Embedded microRNAs and the hypothesized ceRNA mechanism mentioned earlier may be accounted for by such duplication events, as modulating copy number of an embedded microRNA or target site would allow for fine-tuned regulation [56, 65].

### Computational methods for lincRNA target prediction

There have been many attempts to computationally identify the function of lincRNAs. Given the length of lincRNA sequences and the complexity of their potential 3D structures along with the RNA and protein partners, this is a very challenging task. We review the different computational approaches in the following.

#### Correlation with protein coding genes and biological processes

One of the simplest approaches to determine the function of lincRNAs is to examine their correlations with protein coding genes [66]. However, this is a “black box” approach that identifies neither causality nor lincRNA functions at the molecular level. Another naïve approach is to relate the function of lincRNAs to the nearby protein coding genes [67]. Many lincRNAs have been found to exert regulatory activity on protein coding genes in *cis* [45, 52]. However, Khalil et al. found that knockdown of six different lincRNAs did not affect the expression of level of nearby genes [4]. This suggests that lincRNAs can work in *trans* as well, and that the correlation between a lincRNA and its nearby protein coding genes may not necessarily be a causative relationship, but rather a result of sharing a region of active transcription.

#### Relation between lincRNAs with microRNAs and other small non-coding RNAs

Other more sophisticated tools have been developed to identify more succinct functions. Boerner and McGinnis constructed a pipeline to seek functions of lncRNAs in *Zea Mays* [33]. Using BLAST search, they found that the majority of lncRNAs have strong homology to small RNA molecules. They hypothesized that many lncRNAs are simply unprocessed pre-cursors to small non-coding RNAs, such as microRNA, shRNA and siRNA [33]. Based on the “ceRNA hypothesis” mentioned earlier, Liu et al. developed “linc2GO”, a software for identifying mRNA and lincRNA pairs [57]. Using predicted microRNA targets from miRanda, TargetScan and PITA software, they predicted microRNA targets on both mRNAs and lincRNAs; The mRNAs and lincRNAs that had statistically significant target sites for a particular microRNA were proposed to have a “competing endogenous” relationship.

#### Machine learning approaches to target and functional prediction

Machine learning methods have been used successfully to classify whether transcripts are coding or non-coding. However, machine learning methods to identify the targets of lincRNAs have not seen much success. Comparatively, there has been much more success in using supervised learning approaches to identify microRNA targets, such as TargetScan [68], SvMicrO [69] and mirMark [70]. Still progress is being made towards lincRNA functional prediction. Glazko et al. used support vector machines (SVM) to predict lncRNA and PRC2 binding using human lincRNA associated with PRC2 as training data. With the classification model, they were able to predict 59.4 % of lncRNAs which bind to PRC2 in mice [71]. The model was based off of the dataset by Khalil et al. [4, 72] which found roughly 20 % of lincRNAs to associate with PRC2. However, it remains unclear whether the associations were spurious or led to sequence specific chromatin regulation.

#### LincRNA functional prediction through the higher-order structure

Perhaps the least explored lincRNA prediction approach is functional prediction through tertiary and quaternary structure. As the structure of RNA molecules are related to their functions, predicting the structure of complexes between RNA-RNA, and RNA-protein interactions could elucidate functional properties. Several RNA-RNA interaction prediction tools are available, usually based on free-energy, such as RNAhybrid [73] and RNADuplex [74]. RNA-protein interaction prediction tools exist as well, such as RPIseq which uses a Random Forest classification approach [75] or RNApred, which uses an SVM approach [76]. However, there have not been many attempts for lincRNA functional prediction. Many of the protein complexes interacting with lincRNAs do not fall into common binding motifs [41]. Furthermore, functional prediction is complicated by the “n-body problem”, since protein, RNA and DNA can be complexed with lincRNAs simultaneously.

#### Downstream target prediction through directed graphs

Reverse engineering of gene regulatory networks has been an area of research before the explosion of next generation sequencing and lincRNA research [77]. Approaches such as Bayesian networks, information-theoretic approaches and ordinary differential equations have shown strong performance [78]. Generally, a perturbation of the system (such as gene knockout, overexpression or drug treatment) is performed which forces a node (i.e., a gene) on a regulatory network graph to be forcibly turned on or turned off. This perturbation produces direct causative (rather than correlative) downstream effects that can be captured through microarrays and quantitative methods. Recently, Jiang et al. published a database (lncRNA2Target) describing lncRNA knockdown and overexpression experiments, followed by gene quantification by microarray or qPCR [79]. These types of experiments can be a valuable resource for elucidating a lincRNA’s targets and pathways.

## Conclusion

Statistical evaluation studies for lincRNAs are urgently needed, as datasets produced by these various methods have thus far shown only modest overlaps in their identified lincRNAs [14]. Besides lack of sequence conservation among lincRNAs, another major issue hindering functional prediction is the lack of validated data. While there are many well-studied lincRNAs, there are massively more unannotated lincRNAs. Machine learning methods often require a large training dataset to produce accurate results. Several functional lincRNA/lncRNA databases exist (such as lncrnaDB), however the number of entries are very low and do not categorize the function of the lncRNAs in a systematic manner [80]. As more and more lincRNAs become functionally validated, comprehensive and regularly updated databases would be a great source to build good prediction methods. Perhaps even more important is the advancement of experimental techniques to provide quality data required for the prediction. Currently, most experimental techniques focus on a single protein or a small number of proteins (protein-centric) or a single lincRNA or family of lincRNAs (RNA-centric) [41]. New methods are required that can provide high-throughput protein and RNA targets of thousands of lincRNAs in parallel.
